# Supplementary material for: Internal water circulation mediated synergistic co-hydrolysis of PET/cotton textile blends in gamma-valerolactone
Source: Nat Commun. 2024 May 27;15:4498. doi: 10.1038/s41467-024-48937-3 (PMC11130221; doi:10.1038/s41467-024-48937-3)
Supplement: Supplementary file 1 — Supplementary Information [file 41467_2024_48937_MOESM1_ESM.pdf]

Supplementary Information

Internal Water Circulation Mediated Synergistic Co-hydrolysis of PET/cotton Blends Textiles in Gamma-valerolactone

Shun Zhang[1], Wenhao Xu[1], Rongcheng Du[1], Lei Yan[1], Xuehui Liu[2], Shimei Xu[1*], Yu-Zhong Wang[1]

1 Collaborative Innovation Center for Eco-Friendly and Fire-Safety Polymeric Materials (MoE), State Key Laboratory of Polymer Materials Engineering, National Engineering Laboratory of Eco-Friendly Polymeric Materials (Sichuan), College of Chemistry

Sichuan University, Chengdu 610064, China

2 Collaborative Innovation Center for Eco-Friendly and Fire-Safety Polymeric Materials (MoE), State Key Laboratory of Polymer Materials Engineering, National Engineering Laboratory of Eco-Friendly Polymeric Materials (Sichuan), College of Architecture and Environment

Sichuan University, Chengdu 610064, China

* Correspondence author: E-mail: xushimei@scu.edu.cn

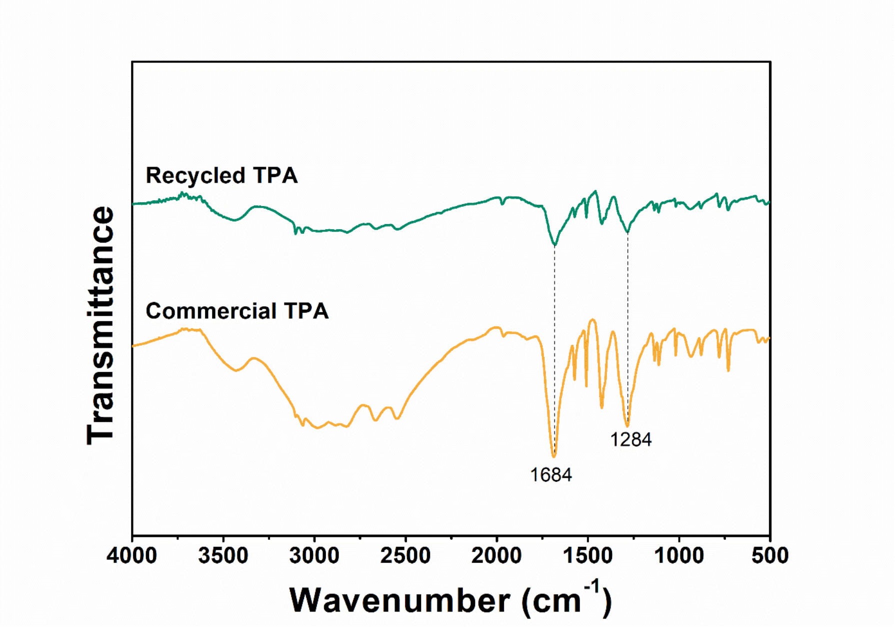

Supplementary Fig. 1 FT-IR spectra of recycled TPA from polyester-cotton blends (80% polyester, 20% cotton).

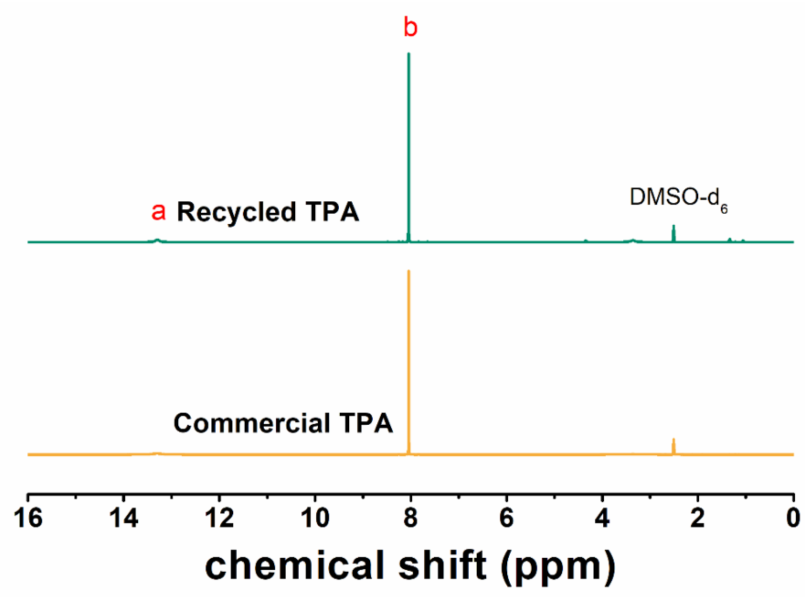

Supplementary Fig. 2 NMR spectra of recycled TPA from polyester-cotton blends (80% polyester, 20% cotton).

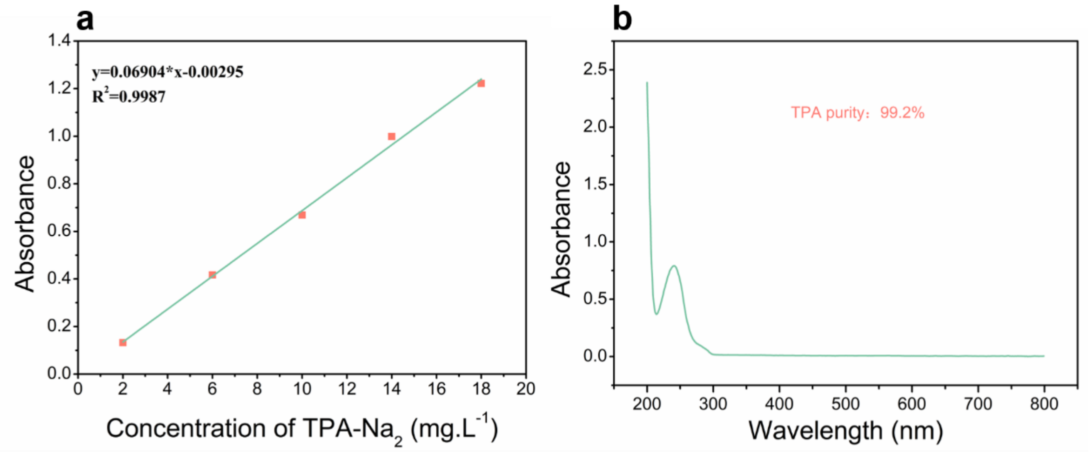

Supplementary Fig. 3 Standard curve of TPA-Na_2 (a), and UV-Vis absorption spectrum of TPA-Na_2 (b) after treatment of recycled TPA with sodium hydroxide according to the reported method.

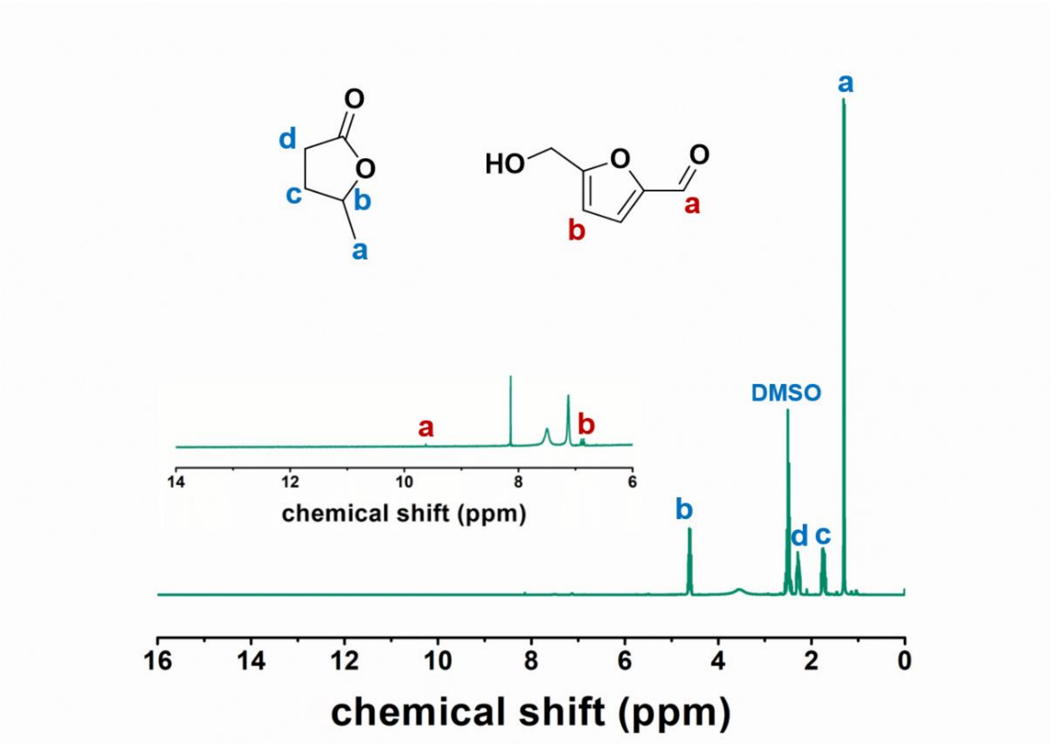

Supplementary Fig. 4 NMR spectra of reaction solution from polyester/cotton blends.

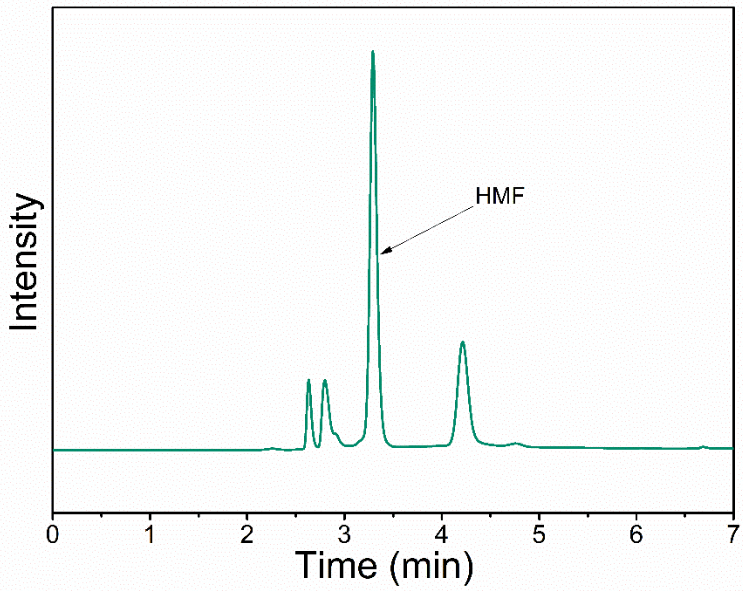

Supplementary Fig. 5 HPLC of reaction solution derived from cotton.

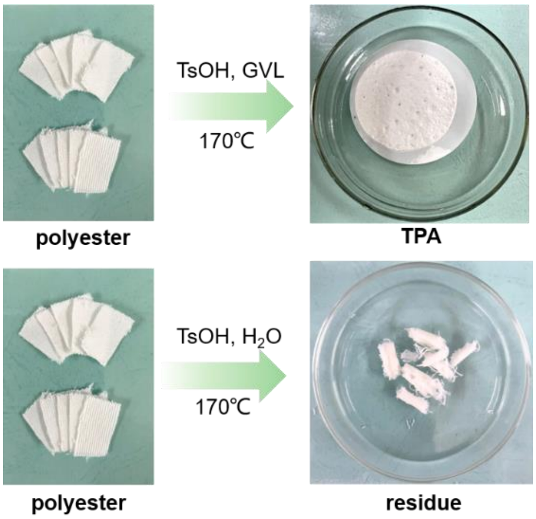

Supplementary Fig. 6 The degradation reaction of polyester in GVL and water.

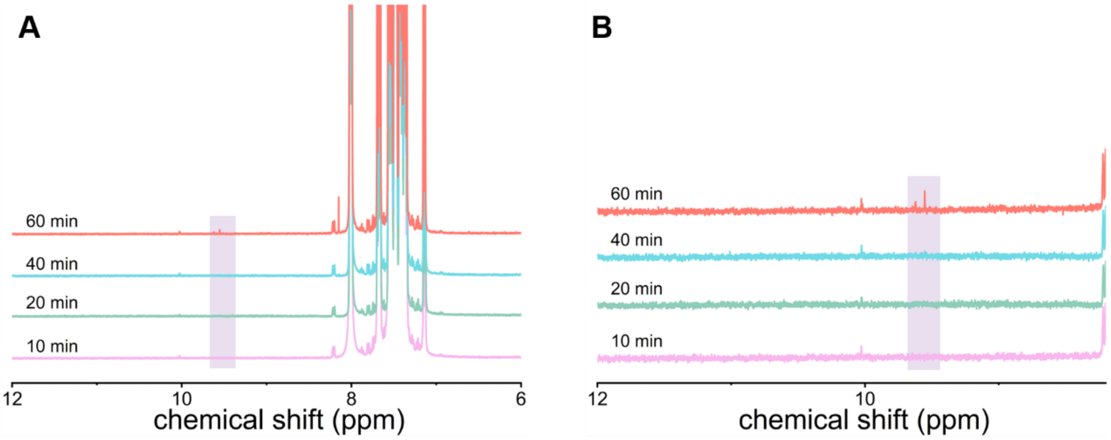

Supplementary Fig. 7 NMR spectra of reaction solution derived from polyester/cotton blends with different time in water (A). NMR spectra with local magnification (B).

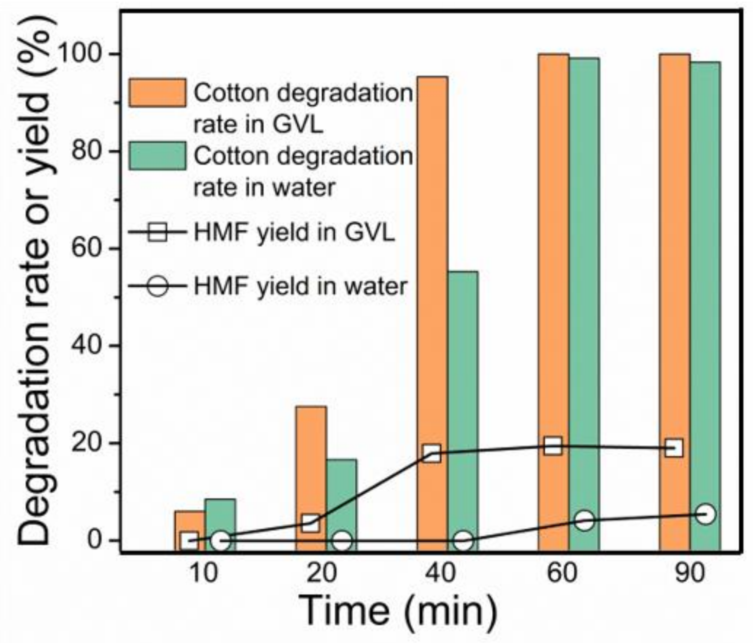

Supplementary Fig. 8 Comparison of HMF production derived from cotton in GVL and water. Reaction under 170 °C with 7.5 wt% TsOH, the GVL is 20 mL.

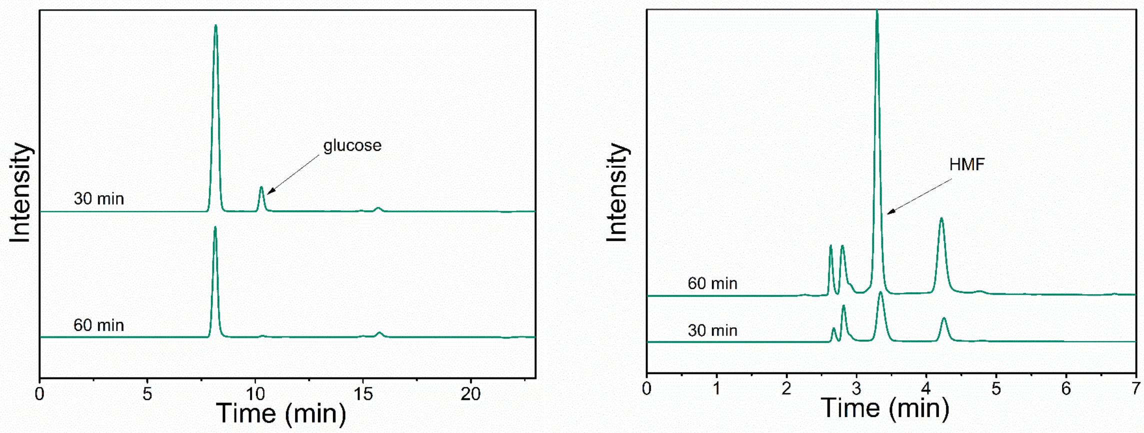

Supplementary Fig. 9 HPLC of reaction solution derived from cotton in GVL at different time.

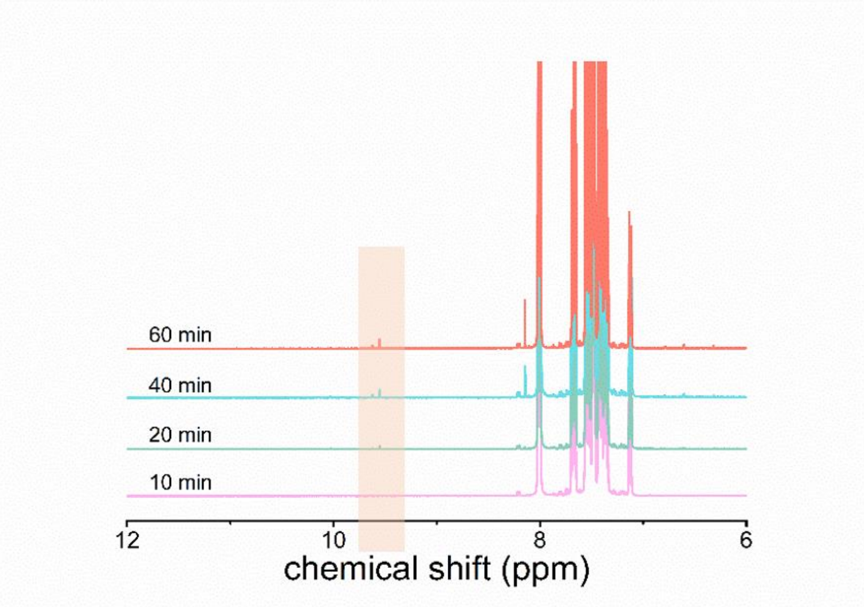

Supplementary Fig. 10 NMR spectra of reaction solution derived from polyester/cotton blends with different time in GVL.

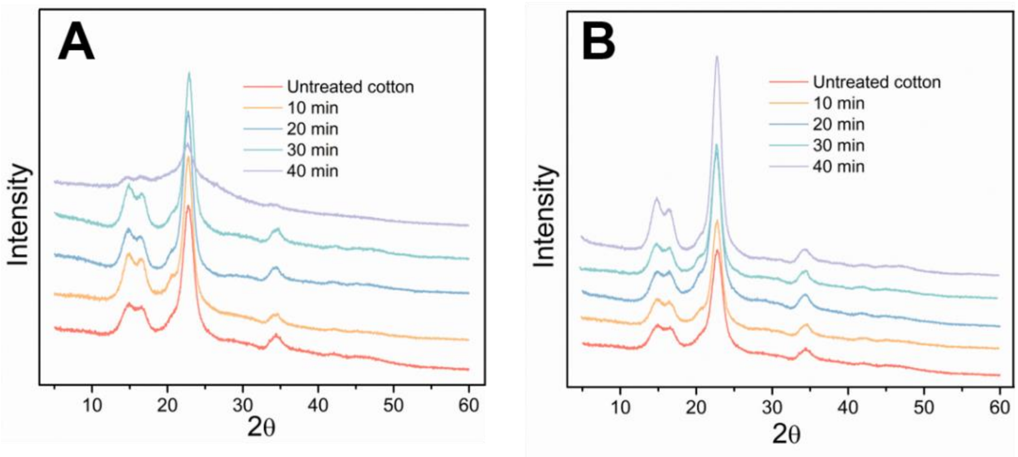

Supplementary Fig. 11 XRD spectra of cotton in GVL(A) and water (B) at different reaction time.

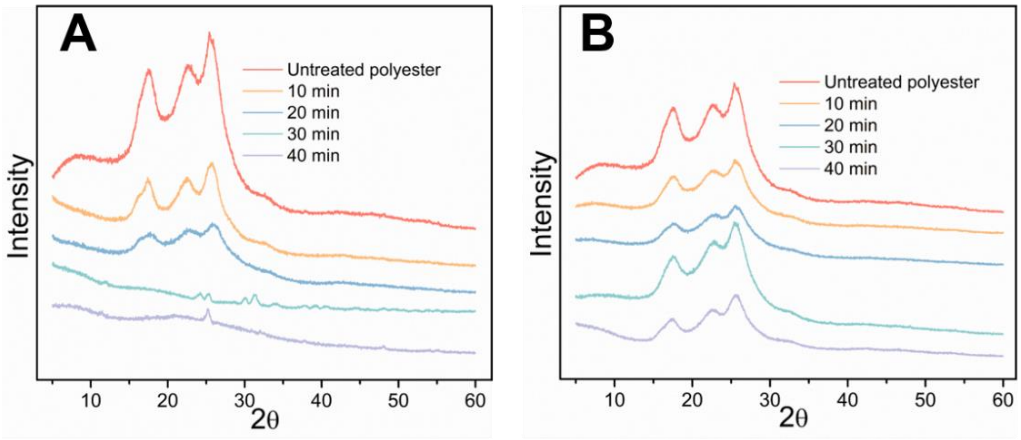

Supplementary Fig. 12 XRD spectra of polyester in GVL(A) and water (B) at different reaction time.

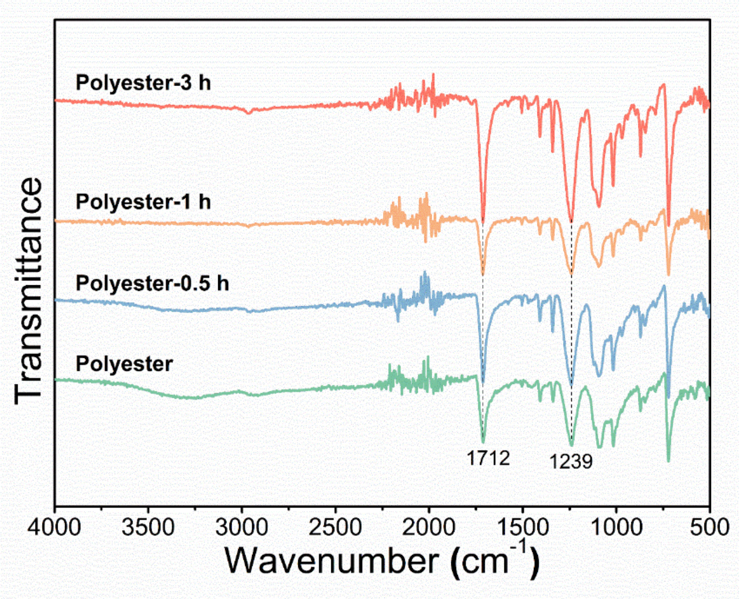

Supplementary Fig. 13 FT-IR spectra of polyester treated with GVL at different time.

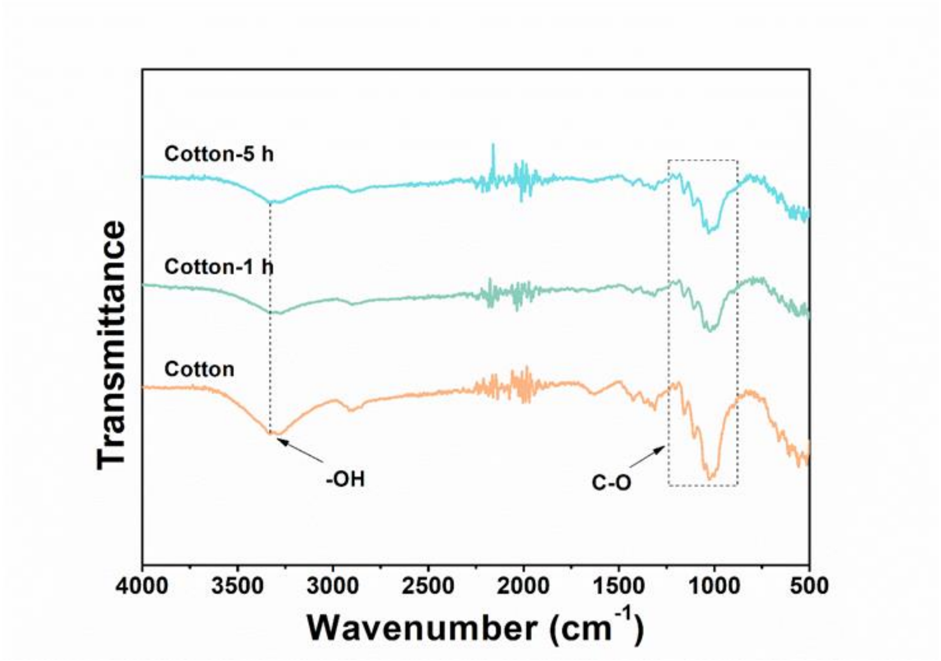

Supplementary Fig. 14 FT-IR spectra of cotton treated with GVL at different time.

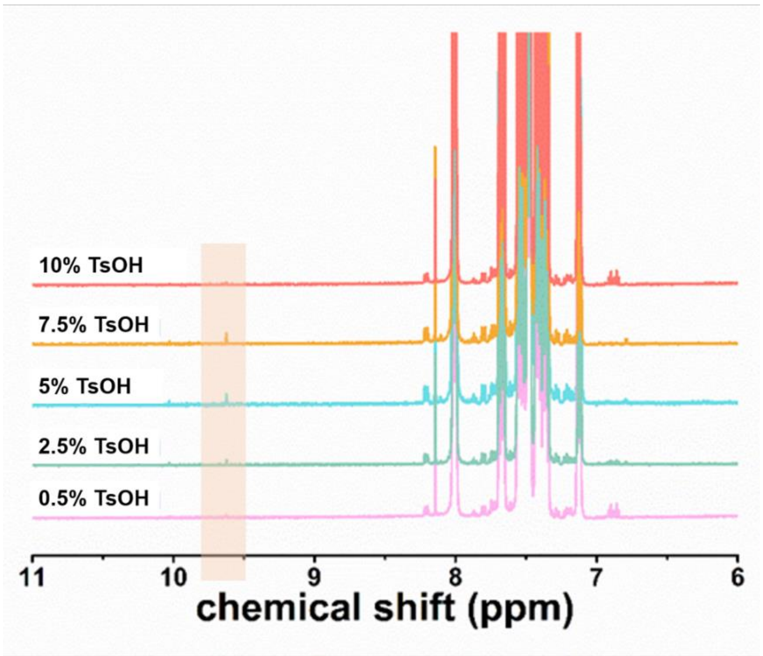

Supplementary Fig. 15 NMR spectra of reaction solution derived from polyester/cotton blends with different amounts of TsOH.

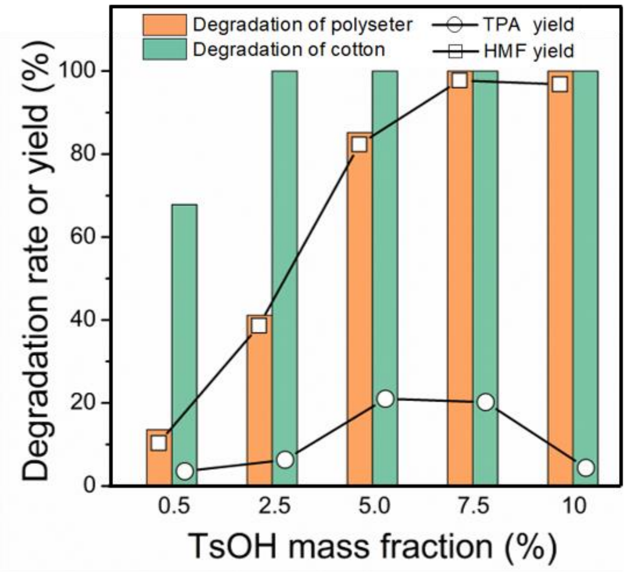

Supplementary Fig. 16 Effect of TsOH dosage on the degradation of polyester/cotton blends. Reaction under 170 °C in 20 mL GVL.

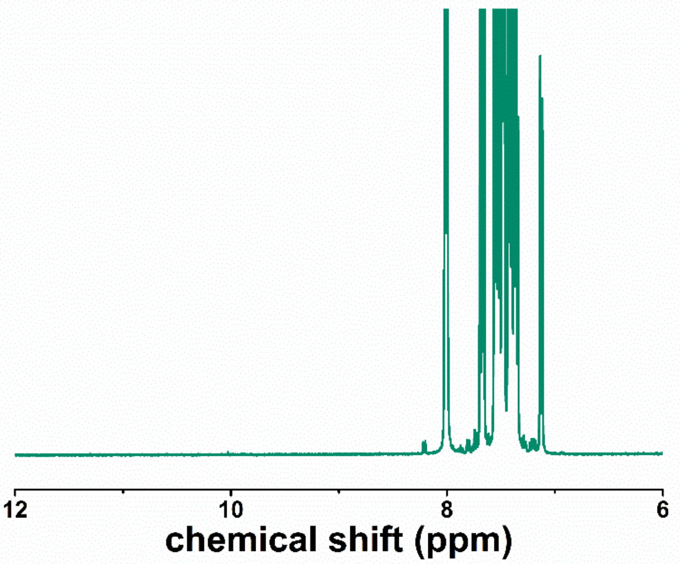

Supplementary Fig. 17 NMR spectra of reaction solution derived from cotton in GVL catalyzed by TPA.

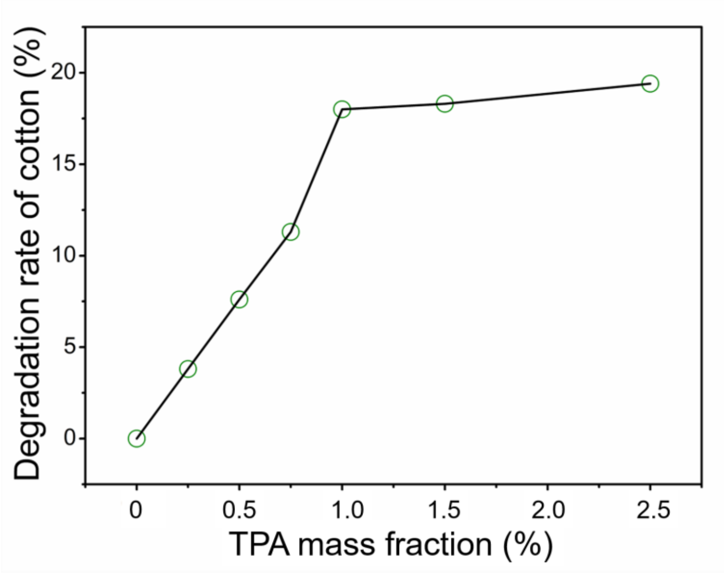

Supplementary Fig. 18 Effect of different amount of TPA on cotton degradation. Reaction conditions: cotton was added to 20 mL GVL solution, in which the mass fraction of cotton was 1%, certain amount of TPA was added at 170 ^{o}C for 1 h.

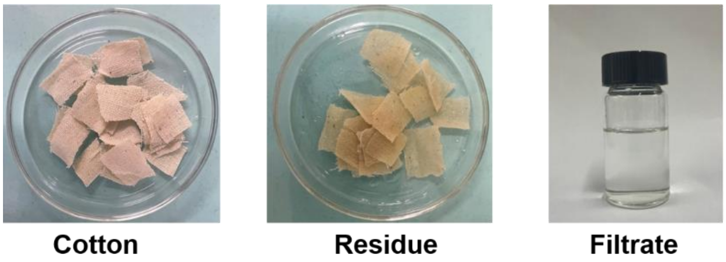

Supplementary Fig. 19 Effect of EG on cotton degradation. Reaction conditions: 1% cotton, mass ratio of cotton to EG of 1:1.3, 170 ºC, 1 h.

Supplementary Table 1. Depolymerization of polyester/cotton blends and HMF yield. [a]

Entry	Time (min)	5-HMF yield (%) [b]	Degradation rate (%)	
			Cotton	Polyester
1	10	0	6.2	0
2	20	1.9	21.2	8.6
3	30	13.5	52.8	23.5
4	40	20.7	97.8	47.8
5	60	24.8	100	100

Polyester and cotton were cut from textiles; [a] 1 g polyester/cotton blends (with a polyester to cotton mass ratio of 8:2), reaction under 170 °C for 60 min with 7.5 wt% TsOH, the GVL is 20 mL; [b] Yield determined by NMR and HPLC.

Reference

1. Zhao Y, Truhlar DG. The M06 suite of density functionals for main group thermochemistry, thermochemical kinetics, noncovalent interactions, excited states, and transition elements: two new functionals and systematic testing of four M06-class functionals and 12 other functionals. *Theoretical Chemistry Accounts* 120, 215-241 (2008).
2. Schafer A, Huber C, Ahlrichs R. Fully Optimized Contracted Gaussian-basis Sets of Triple Zeta Valence Quality For Atoms LI To KR. *Journal of Chemical Physics* 100, 5829-5835 (1994).
